# Assessing the Quality of Hearing Aids-Related Videos on TikTok

**DOI:** 10.3389/fpubh.2022.901976

**Published:** 2022-06-22

**Authors:** Kai Chen, Li Zhou, Rui Zhao, Yuedi Tang

**Affiliations:** ^1^Department of Otorhinolaryngology, West China Hospital of Sichuan University, Chengdu, China; ^2^Department of Otorhinolaryngology, Hospital of Chengdu University of Traditional Chinese Medicine, Chengdu, China; ^3^Core Facilities of West China Hospital, Chengdu, China

**Keywords:** hearing aids, hearing loss, internet health information, information quality, social media

## Abstract

Hearing aids are effective at improving listening ability and health-related quality of life. Recently, we observed that there are many hearing aids-related videos published on TikTok. However, the quality of the information they offer remains unstudied. This study aimed to evaluate the information quality of hearing aids videos on TikTok. We collected a sample of 155 hearing aids-related videos in Chinese and extracted the basic information. First, we identified the source of each video. Two independent raters assessed the quality of the information in the videos, using the PEMAT-A/V tool and DISCERN instrument. Regarding content, the results showed that the video contents on TikTok mainly about features, functionalities, and suggestions of purchase or fitting of hearing aids, while the information about the disadvantages and complications of hearing aids was limited. The overall quality of the hearing aids-related videos was acceptable on average, although the quality varies greatly depending on the type of source. Patients should be cautious in obtaining information about hearing aids on TikTok.

## Introduction

Acquired hearing loss is common and increased significantly with age (1). People with hearing impairment may experience self-recognized hearing loss or family concerns (2). They observe difficulty in communication, often asking others to repeat things, social avoidance, and hearing difficulties in the context of background noise (2). Hearing aids amplify the sound and make use of the residual hearing so that the sound can be sent to the auditory center of the brain and feel the sound. Multiple studies have shown that hearing aid is beneficial to patients with hearing loss (2, 3). Hearing aids are effective at improving hearing-specific health-related quality of life, general health-related quality of life, and listening ability in adults with mild to moderate hearing loss (4). As a result of universal newborn hearing screening, infants with hearing loss have earlier access to intervention, which has a positive impact on the speech and language outcomes of young children (5, 6). As for children, increased audibility provided by hearing aids influences language outcomes, if children wear their devices on a consistent basis (7).

With the popularity of social media, access to information has become more and more diverse, which have an unprecedented influence on people's daily life. People who watch online videos account for 85% of Internet users in the USA, while it is as high as 92% in China (8). Compared with other online sources, short video applications can offer rich technology features and useful social media affordances to users, such as beauty and makeup, education, cooking, wellness and technology, and educational healthcare content has become an important part of TikTok's content ecosystem (8). The world's most popular short video app, TikTok, and its Chinese version reached first place on the global mobile app download list, with one billion active monthly users (8, 9). In TikTok, short videos can attract users through salient and engaging contents and images, and stimulate users' buying desire. Especially in the application of the Chinese version, users can directly jump to the product introduction and shopping page. Although medical products cannot be directly purchased on TikTok currently, the attraction and diversion of short videos to patients cannot be ignored. In addition, users may share the information they found with friends, family, and discussion purposes (10).

Song et al. found that hedonic social applications such as TikTok are an important channel for users to access health information (11). TikTok affords rich information modalities and contains ample technology features such as commenting, chatting, liking, following, and live-streaming, which make the app easier for the users to use as a source of health information (11). TikTok learns quickly via artificial intelligence what users like (12), which results in more recommendations of hearing aids-related videos for users with hearing impairment. Many health professionals and medical institutions have tried to distribute health knowledge and promote public health literacy on short video applications, such as TikTok, which demonstrated a vast potential for disseminating health information (8, 13).

Despite the benefits of social media, its use for health communication has some shortcomings (14). Information quality is one of the most significant concerns when users seek health information online (15). Emerging technologies provide great health communication opportunities that can provide information about hearing aids for hearing loss patients. When ordinary people receive wrong or misleading information, they may make wrong decisions based on it (16). Therefore, it is important to examine the quality of hearing aids-related videos on TikTok.

We observed that there are many hearing aids-related videos on TikTok. However, the quality of the information they offer remains unstudied. Therefore, to fill this gap, this study aimed to evaluate the information quality of hearing aids videos on TikTok.

## Methods

### Search Strategy and Data Extraction

**Figure 1:**
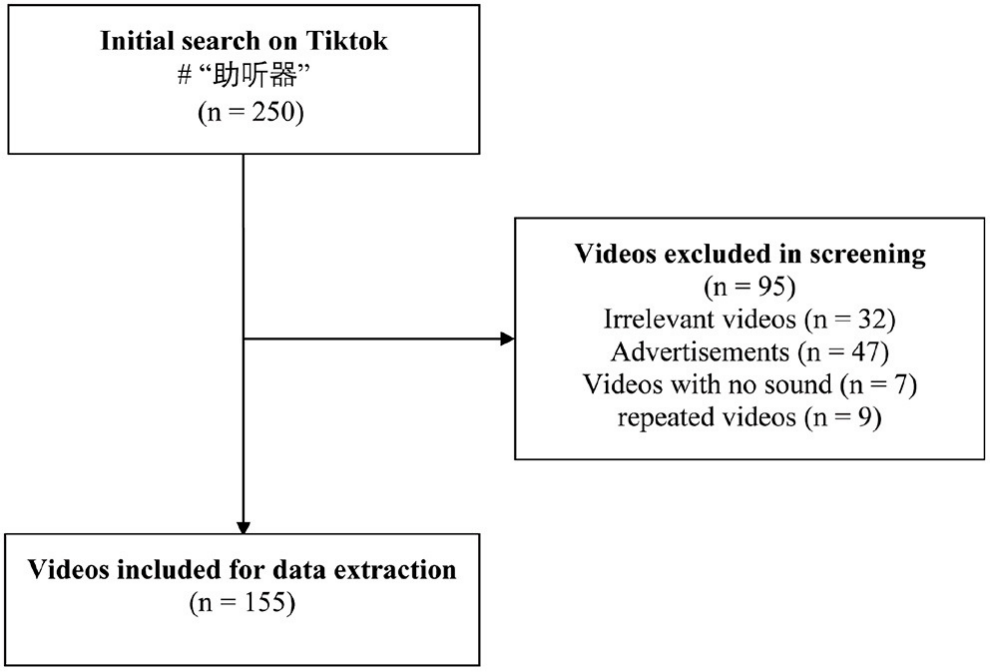
Video screening procedure.

Videos were searched on TikTok on March 16, 2022, using the keyword “助听器” (“hearing aids” in Chinese). TikTok search was conducted on a newly installed app on an iPhone 13 (v.15.3) with a cleared cache and without any login. TikTok provides three ways to sort items: “overall ranking”, “most recent”, and “most likes”. Given that most users employ the overall ranking mode, the default mode of sorting recommended by TikTok, we used this mode to retrieve the first 250 videos. We included videos directly related to hearing aids, excluding commercial videos and duplicate videos. After screening, we obtained 155 videos for data extraction and analysis, representing 62% (Figure 1). We extracted the basic information of each included video, including URL, release date, uploader name, uploader type (individual or organization), uploader verification status, video length, and the number of “likes” and comments it received.

### Quality Assessment

We measured 2 aspects of hearing aids-related videos on TikTok: their understandability, actionability, and reliability. First, to rate the understandability and actionability of the videos, we adopted the Patient Education Materials Assessment Tool for Audio-Visual Materials (PEMAT-A/V) tool (17). PEMAT is a systematic method to evaluate and compare the understandability and actionability of patient education materials. It is designed as a guide to help determine whether patients will be able to understand and act on information. PEMAT-A/V for audiovisual materials consisting of 13 items measuring understandability and 4 items measuring actionability. Except for Not Applicable (N/A) items, each item will be given either 1 point (Agree), or 0 points (Disagree). Divide the sum by the total possible points, excluding the items that were scored Not Applicable (N/A). Multiply the result by 100 and you will get a percentage (%). This percentage score is the understandability or actionability score on the PEMAT. Scores under 70% indicate that the information had poor understandability or actionability.

We adopted the DISCERN instrument to rate the reliability of eligible videos (18). DISCERN is a reliable and valid instrument for judging the quality of written consumer health information (18). Although the original DISCERN instrument was designed for evaluating written publications, it has been widely used for assessing health-related videos (14). DISCERN instrument consists of 15 questions plus an overall quality rating, with response choices based on a 5-point scale, ranging from 1 = poor to 5 = good. Due to the hearing aids-related videos on TikTok is mostly focused on the popularization of science, we only chose the reliability of the videos to rate. The scores for reliability were reported as percentages (%).

Two independent researchers (KC and LZ) evaluated and rated the videos. A third researcher (YT) helped to resolve the discrepancies between the two reviewers.

### Statistical Analysis

SPSS 21.0 statistical software (SPSS, Inc., Chicago, USA) was used for data analyses. Shapiro-Wilk test was used to assess the normality of data. The interrater reliability (Cohen κ) for each item ranged from 0.902 to 0.942 (*P* < 0.001). These results indicated that the rating process had satisfactory interrater reliability. Medians and interquartile ranges (IQRs) were used to analyze as the DISCERN and PEMAT-A/V scores data failed the Shapiro–Wilk normality tests. Any analyses of scores based on video sources were performed using the Kruskal-Wallis H test. A two-tailed *p*-value of <0.05 was considered to be statistically significant.

## Results

### Video Characteristics

Among the screened videos, they were released from December 16, 2019, to March 15, 2022. The shortest video last 16 s and the longest video last 485 s. The median video duration was 81 s. The videos in the sample received 52,568 “likes” and 4,143 comments.

The hearing aids videos on TikTok came mainly from two types of sources: individual users and organizational users. Individual users published most of the videos (*n* = 106, 68.3%). Among individual users, health professionals created the most videos (*n* = 76, 49%), followed by science communicators (*n* = 23, 14.8%) and general users (*n* = 7, 4%). meanwhile, the hearing aid fitters division accounts for 77.6% of the health professionals. Among organizational users, for-profit organizations published the most videos (*n* = 42, 27%), followed by non-profit organizations (*n* = 4, 2%) and news agencies (*n* = 3, 1.9%). The characteristics of the videos were shown in Table 1.

**Table 1:** Characteristics of the videos (median and interquartile ranges).

**Source type**	**Length of video (seconds), median (IQR)**	**“Likes”, median (IQR)**	**Comments, median (IQR)**
Health professionals^a^ (*n* = 76)	67 (46.25–119.5)	54.5 (23–171.75)	8 (1–23.5)
Science communicators^b^ (*n* = 23)	111 (82.5–144.5)	15 (7.5–98.5)	1 (0–10.5)
General users^c^ (*n* = 7)	82 (59–126)	81 (65–191.5)	23 (15–54.5)
For-profit organizations^d^ (*n* = 42)	82.5 (55.25–125.25)	23.5 (9.25–57.25)	4 (1–7.75)
Non-profit organizations^e^ (*n* = 4)	100 (97.75–107.25)	103 (57–165)	6 (2.5–13.25)
News agencies (*n* = 3)	109 (93–125)	144 (72.5–270)	4 (2–7.5)
Total (*n* = 155)	81 (53.5–128.5)	41 (12–116)	6 (1–21)

a *Health professionals were individuals who identify themselves as health professionals (e.g., doctors and hearing aid fitters)*.

b *Science communicators were individuals who are engaged in scientific communication*.

c *General users: consumers*.

d *For-profit organizations were organizations that pursue commercial interests*.

e *Non-profit organizations were organizations operated for social benefit and public hospitals*.

**Figure 2:**
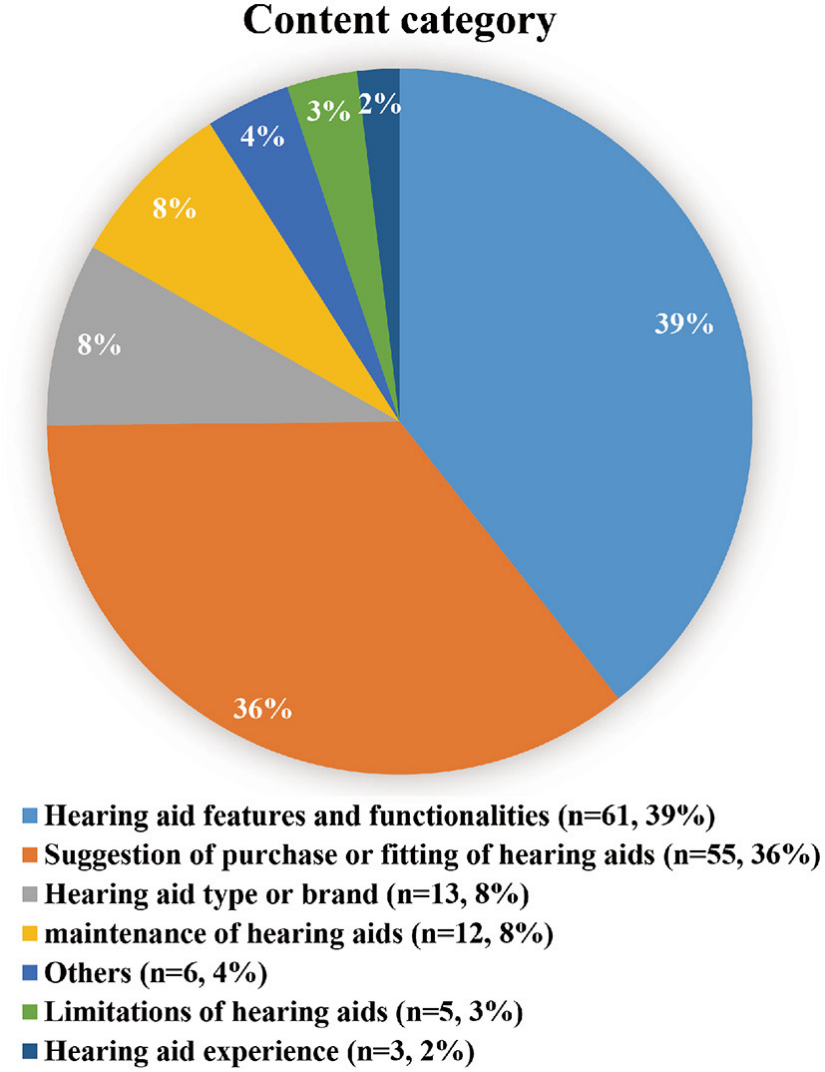
Distribution of video content.

The contents of hearing aids videos on TikTok were mainly about two aspects: hearing aid features and functionalities (39.3%) and suggestion of purchase or fitting of hearing aids (35.4%). Then comes hearing aids type or brand (8.3%) and hearing aids maintenance (7.7%). The content category of the videos was shown in Figure 2.

### Information Quality

Regarding reliability, videos released by non-profit organizations (median = 57.5%, IQR = 57.5–61.88%) had the highest scores, while those from general users (median = 37.5%, IQR = 32.5–40%) were lowest. Our results showed that news agencies (median = 57.5%, IQR = 56.25–60%) and health professionals (median = 47.5%, IQR = 41.88–60%) also contributed videos with relatively high reliability. For-profit organizations (median = 42.5%, IQR = 37.5–47.5%) and science communicators (median = 42.5%, IQR = 40–47.5%) contributed videos with relatively low reliability. The differences in reliability across the different video sources were statistically significant (Table 2).

**Table 2:** DISCERN and PEMAT-A/V scores of diabetes-related TikTok videos by source.

**Video source**	**Reliability (DISCERN) (%), median (IQR)**	**Understandability (%), median (IQR)**	**Actionability (%), median (IQR)**	**Total PEMAT-A/V scores (%), median (IQR)**
Health professionals (*n* = 76)	47.5 (41.88–60)	87.5 (75–88.89)	66.67 (33.33–100)	81.82 (72.73–85.23)
Science communicators (*n* = 23)	42.5 (40–47.5)	87.5 (70–95)	33.33 (33.33–66.67)	75 (64.29–83.97)
General users (*n* = 7)	37.5 (32.5–40)	66.67 (50–77.78)	0 (0–50)	50 (37.5–75)
For-profit organizations (*n* = 42)	42.5 (37.5–47.5)	77.78 (75–88.89)	66.67 (33.33–66.67)	75 (64.39–83.33)
Non-profit organizations (*n* = 4)	57.5 (57.5–61.88)	100 (97.22–100)	50 (33.33–75)	83.97 (83.33–88.46)
News agencies (*n* = 3)	57.5 (56.25–60)	88.89 (88.89–94.44)	33.33 (33.33–50)	75 (75–83.33)
Total (*n* = 155)	45 (40–52.5)	87.5 (75–88.89)	66.67 (33.33–66.67)	75 (66.67–88.33)
*P*-value^a^	<0.001	0.007	0.004	0.018

a *P-values were calculated with the Kruskal-Wallis H test*.

Videos published by non-profit organizations (median = 100%, IQR = 97.22–100%) had the highest understandability, whereas the videos contributed by general users (median = 66.67%, IQR = 50–77.78%) had the lowest reliability. News agencies (median = 88.89%, IQR = 88.89–94.44%), science communicators (median = 87.5%, IQR = 70–95%), and health professionals (median = 87.5%, IQR = 75–88.89%) also contributed videos with relatively high reliability. For-profit organizations (median = 77.78%, IQR = 75–88.89%) contributed videos with relatively low reliability. The differences in understandability across the different video sources were statistically significant (Table 2).

Regarding actionability, videos released by the health professionals (median = 66.67%, IQR = 33.33–100%) had the highest scores, while those from the general users (median = 0%, IQR = 0–50%) were lowest. Our results showed that the for-profit organizations (median = 66.67%, IQR = 33.33–66.67%) and the non-profit organizations (median = 50%, IQR = 33.33–75%) also contributed videos with relatively high reliability. The differences in reliability across the different video sources were statistically significant (Table 2).

With regard to the last item concerning the total PEMAT-A/V scores, the highest-quality videos were created by non-profit organizations (median = 83.97%, IQR = 83.33–88.46%) and the lowest-quality videos were generated by general users (median = 50%, IQR = 37.5–75%). The differences were statistically significant (Table 2).

## Discussion

### Principal Findings

This study systematically evaluated the information quality of hearing aids-related videos on TikTok. With the development of the Internet, various social media channels provide convenient means for patients to seek medical knowledge. Our research found that TikTok is an important platform for information-related hearing aids. The 155 videos we studied received 52,568 “likes” and 4,143 comments, which indicates that TikTok is a promising channel for health communication.

In the present study, we use the classification of previous research to divide the uploaders into individual users and organizational users (14). Individual users include health professionals, science communicators, and general users, and organization users include for-profit organizations, non-profit organizations, and news agencies. Similar to the previous study based on YouTube (19), our study suggested that health professionals provide more videos, followed by for-profit organizations. This indicates that health professionals were committed to promoting hearing aids knowledge on TikTok in China, while news agencies use this channel less frequently. It's important to note that most health professionals are hearing aid fitters, which is an important publicity means for profit organizations.

In terms of video content, the study found that most of the videos were about the features and functionalities, and purchase or fitting suggestions, while few videos introduced the disadvantages or complications of hearing aids. These results are consistent with a previous study, whose content categories with over 50% of all videos commenting on general information about hearing aids, hearing aid types, and handling and maintenance of hearing aids (19). Secondly, most of the videos do not involve other therapies except hearing aids in patients with hearing loss. They often take hearing aids as the most important and effective treatment choice for hearing loss patients in their videos, especially in the videos published by hearing aid fitters. This problem usually doesn't appear in the ear, nose, and throat doctors' videos. Given the observed imbalance of video content, we suspect that the current video related to hearing aids on TikTok cannot fully meet the information needs of patients. Therefore, we call for more relevant videos to address the comprehensive information needs of patients.

Our research found that the information quality of videos was mixed. Overall, the videos published by non-profit organizations and health professions had a high quality, while those from the for-profit organizations had the lowest quality. Given the uneven quality of videos, we recommend that patients be cautious in obtaining information about hearing aids on TikTok, avoiding unnecessary hearing loss caused by wrong information.

### Limitations and Future Directions

This research should be viewed in light of several limitations. Firstly, we only include hearing aids-related videos in our study in Chinese. We hope that more studies in the future to evaluate the information quality of hearing aids-related videos in other languages on TikTok. Furthermore, our study does not discuss the misinformation of videos, and future research needs to discuss the influence of misinformation on patients.

## Conclusion

This study evaluated the information quality of 155 hearing aids-related videos on TikTok. The results show that the video contents on TikTok mainly about features, functionalities, and suggestions of purchase or fitting of hearing aids, while the information about other aspects was limited, such as the disadvantages and complications of hearing aids. The overall quality of the hearing aids-related videos was found to be acceptable on average, although the quality varies greatly depending on the type of source. Patients should be cautious in obtaining information about hearing aids on TikTok.

## Data Availability Statement

The raw data supporting the conclusions of this article will be made available by the authors, without undue reservation.

## Author Contributions

KC: design, conduct, analyzed data, and drafting. LZ: conduct and analyzed data. RZ: conduct. YT: design of the study, revised article, final approval, and accountable for all aspects. All authors contributed to the article and approved the submitted version.

## Funding

The work was funded by the Science and Technology of Department of Sichuan Province (No.: 2021YJ0183).

## Conflict of Interest

The authors declare that the research was conducted in the absence of any commercial or financial relationships that could be construed as a potential conflict of interest.

## Publisher's Note

All claims expressed in this article are solely those of the authors and do not necessarily represent those of their affiliated organizations, or those of the publisher, the editors and the reviewers. Any product that may be evaluated in this article, or claim that may be made by its manufacturer, is not guaranteed or endorsed by the publisher.
